# Two salivary proteins Sm10 and SmC002 from grain aphid *Sitobion miscanthi* modulate wheat defense and enhance aphid performance

**DOI:** 10.3389/fpls.2023.1104275

**Published:** 2023-03-28

**Authors:** Yu Fu, Xiaobei Liu, Qian Wang, Huan Liu, Yumeng Cheng, Hongmei Li, Yong Zhang, Julian Chen

**Affiliations:** ^1^ State Key Laboratory for Biology of Plant Diseases and Insect Pests, Institute of Plant Protection, Chinese Academy of Agricultural Sciences, Beijing, China; ^2^ Ministry of Agricultural and Rural Affairs-Centre for Agriculture and Bioscience International (MARA-CABI) Joint Laboratory for Bio-Safety, Institute of Plant Protection, Chinese Academy of Agricultural Sciences, Beijing, China

**Keywords:** *Sitobion miscanthi*, salivary effectors, wheat, defense responses, aphid performance, bacteria type III secretion system

## Abstract

The grain aphid *Sitobion miscanthi* is a serious pest of wheat that causes severe economic damage by sucking phloem sap and transmitting plant viruses. Here, two putative salivary effector homologs from *S. miscanthi* (Sm10 and SmC002) were selected based on sequence similarity to other characterized aphid candidate effectors. These effectors were then delivered into wheat cells separately *via* the type III secretion system of *Pseudomonas fluorescens* to elucidate their functions in the regulation of plant defenses and host fitness. The results showed that the delivery of either Sm10 or SmC002 into wheat plants significantly suppressed callose deposition and affected the transcript levels of callose synthase genes. The expression levels of salicylic acid (SA)-associated defense genes were upregulated significantly in wheat leaves carrying either Sm10 or SmC002. Moreover, LC-MS/MS analysis revealed that wheat SA levels significantly increased after the delivery of the two effectors. The results of aphid bioassays conducted on the wheat plants carrying Sm10 or SmC002 showed significant increases in the survival and fecundity of *S. miscanthi*. This study demonstrated that the Sm10 and SmC002 salivary effectors of *S. miscanthi* enhanced host plant susceptibility and benefited *S. miscanthi* performance by regulating wheat defense signaling pathways.

## 1 Introduction

Aphids (Hemiptera: Aphididae) are major pests in agriculture that cause serious economic losses to all important cultivated crops worldwide (Ogawa and Miura, 2014). They have piercing-sucking mouthparts that damage plants directly, by depleting nutrients and altering plant development, and indirectly, by supporting growth of the sooty mold fungus and by vectoring numerous plant viruses (Blackman and Eastop, 2000).

During aphid probing and feeding, salivary proteins of aphids are secreted into plants through needle-like mouthparts, which has been demonstrated to be involved in the modulating plant defense responses (Tjallingii, 2006; Will et al., 2007; Hogenhout and Bos, 2011; Elzinga and Jander, 2013). As of now, the identified salivary proteins are mainly divided into two categories: elicitor and effector. Elicitors, subset of salivary proteins, similar to plants pathogen-associated molecular patterns (PAMP), can be recognized by pattern-recognition receptors (PRRs) on the plant and further trigger PAMP-triggered immunity (PTI), or be resisted by plant resistance (R) proteins activating a stronger effector-triggered immunity (ETI) (Jones and Dangl, 2006; Rodriguez and Bos, 2013; Snoeck et al., 2022). For example, green peach aphid *Myzus persicae* salivary proteins, Mp10, Mp42 and Mp58, overexpression in *Nicotiana benthamiana* stimulated an intense immune response in host plant, causing phenotypes such as leaf yellowing and cell necrosis, and reduced the fecundity of *M. persicae* (Bos et al., 2010; Rodriguez et al., 2014). In addition, several secreted effectors of aphid saliva can suppress plant defenses, thus promoting host susceptibility) (Hogenhout and Bos, 2011). For example, C002, a first effector identified from the salivary glands of the pea aphid, *Acyrthosiphon pisum*, is essential for aphid feeding on a host plant, ApC002 knockdown could significantly reduce the aphid feeding duration in the plant phloem (Mutti et al., 2008). MpC002, a homologous protein of ApC002 identified in *M. persicae*, the overexpression enhanced host susceptibility by suppressing plant defenses (Bos et al., 2010; Elzinga et al., 2014). Mp55, another salivary protein of *M. persicae* overexpression in *Arabidopsis thaliana*, also significantly improved aphid reproduction (Elzinga et al., 2014). Transiently overexpressing salivary proteins Me10, Me23 and Me47 of *Macrosiphum euphorbiae* in *N. benthamiana* effectively inhibited the plant immunity and improved the host adaptability of aphids (Atamian et al., 2013; Kettles and Kaloshian, 2016). Expression of candidate effectors RpC002 and Rp1 from the bird cherry-oat aphid *Rhopalosiphum padi* in transgenic barley lines enhanced plant susceptibility to aphids by suppressing plant defense responses (Escudero-Martinez et al., 2020).

Although the role of salivary effectors is highlight in the study of aphid virulence, but few study has been performed to precisely identify the composition salivary proteins of the cereal aphid and the roles of potential effectors in wheat-aphid interactions, mainly due to the poor efficiency of *Agrobacterium*-mediated transformation in cereals (Shrawat and Lörz, 2006). Recently, *Pseudomonas fluorescens* with an engineered bacterial type III secretion system (T3SS) has been established and successfully applied in effectors of wheat aphids by in our research team, for example, delivering candidate effector Sg2204 of greenbug *Schizaphis graminum* into wheat using *P. fluorescens* suppressed wheat SA and JA defense responses and enhanced aphid feeding on host plants (Zhang et al., 2022). The grain aphid *Sitobion miscanthi* is one of the most important and widely distributed cereal aphids, which was previously misusing as English grain aphid *Sitobion avenae* in China, causing huge yield losses by feeding phloem sap and transmitting barley yellow dwarf virus (Blackman and Eastop, 2000; Jiang et al., 2019). Watery saliva of *S. miscanthi* has been previously involved in modulating wheat defense responses (Zhang et al., 2017a). In addition, 526 transcripts predicted to encode secretory proteins and 114 salivary proteins were identified from the salivary glands and watery saliva of *S. miscanthi* using transcriptomic and proteomic analyses respectively, and among them, homologous of Me10 and ApC002 were identified in salivary gland transcriptomes and saliva proteomes of *S. miscanthi* (Zhang et al., 2017b; Zhang et al., 2021). However, whether these homologous effectors from *S. miscanthi* have conserved functions in modulating aphid-wheat interactions are still unclear.

In this study, we selected two putative salivary effectors Sm10 and SmC002 of *S. miscanthi* based on reported RNA-seq data and their sequence similarity to previously characterized aphid effectors, and then detected the function of these two candidate effectors in regulating wheat defense and aphid performance *via* T3SS-mediated transient expression wheat system, providing further insights into the mechanisms on cereal aphid-wheat interactions.

## 2 Materials and methods

### 2.1 Aphid and plants

Clone of the parthenogenetic grain aphid *S. miscanthi* was initially established from single aphid collected from a wheat field in Langfang Experimental Station, located in Langfang city, Hebei Province, northern China, and maintained under laboratory conditions (L: D = 16 h: 8 h, 20 ± 1°C) on wheat plants (cv Mingxian 169) for more than 3 years (25-30 generations per year). Winter wheat (*Triticum aestivum*) cultivars Mingxian169 were grown in a climate chamber at 20°C ± 1°C under 16-h light/8-h dark photoperiod until reaching the two-leaf stage (12 day-old). Tobacco plants *N. benthamiana* were grown in an environmentally controlled greenhouse at 23°C ± 1°C under 16-h light/8-h dark photoperiod. *N. benthamiana* plants were used in transient transformation experiments after 4–5 weeks.

### 2.2 Sequence bioinformatics analysis

The multiple alignment of amino acid sequences was performed using Clustal Omega (RRID : SCR_001591) (https://www.ebi.ac.uk/Tools/msa/clustalo/). A phylogenetic tree was constructed by the neighbor-joining method *via* MEGA7.0 (RRID : SCR_000667). Bootstrap values were calculated as a percentage from over 1000 replications. The signal peptides were predicted using the SignalP 4.1 server (RRID : SCR_015644) (http://www.cbs.dtu.dk/services/SignalP/) and iPSORT (http://ipsort.hgc.jp/). The protein molecular weight was predicted by the Compute pI/Mw tool (http://web.expasy.org/com-pute_pi/) on ExPASy (RRID : SCR_007944). The prediction of transmembrane regions, Pfam domains and nuclear localization signals in protein sequences was performed using TMHMM 2.0 (RRID : SCR_014935) (http://www.cbs.dtu.dk/services/TMHMM-2.0/), Pfam (RRID : SCR_004726) (http://pfam.xfam.org/) and NLStradamus (Nguyen Ba et al., 2009) (http://www.Moseslab.csb.utoronto.ca/NLStradamus/), respectively. The prediction and localization of protein motifs were conducted in MEME (RRID : SCR_001783) (http://meme-suite.org/tools/meme).

### 2.3 Subcellular localization patterns of candidate effectors in *Nicotiana benthamiana*

The ORF sequences of two effectors without an N-terminal signal peptide were cloned into pBWA(V)HS-GLosgfp vector (BioRun, Wuhan, China) with TagGFP fused at the C-terminus using *Eco31*I restriction enzymes (Thermo Fisher Scientific, MA, USA), and all constructs were then transformed into *Agrobacterium tumefaciens* strain EHA105 (Biomed, Beijing, China). After culturing overnight at 28°C, the cells were harvested and resuspended in infiltration buffer (10 mmol/L 2-(N-morpholino) ethanesulfonic acid, 20 mmol/L acetosyringone, and 10mmol/L MgCl_2_, all reagents from Sigma-Aldrich, MO, USA) to reach an optimal density at an OD_600_ of 0.3. The suspensions were kept at room temperature for 4-6 h and then infiltrated into *N. benthamiana* (4-week-old) leaves with a 1 mL syringe without a needle. The infiltrated leaves were collected and observed using a Zeiss LSM 880 laser confocal microscope (Zeiss, Jena, Germany) post four days infiltration. GFP and chlorophyll autofluorescence were excited at 561 and 488nm, respectively, and collected at 586–647 and 680–700nm, respectively. Three biological replicates were performed.

### 2.4 Validation of the signal peptide secretion activity of candidate effectors

Predicted signal peptides of Sm10 and SmC002 were functionally validated using the yeast secretion system as reported previously (Oh et al., 2009). Briefly, the DNA fragments of Sm10, SmC002, Avr1b (positive control) and the Mg47 (negative control) signal peptide were introduced into pSUC2T7M13ORI (pSUC2, provided by Professor Zhongyue Wang from Chinese Academy of Agricultural Sciences) by *EcoR*I and *Xho*I (Thermo Fisher Scientific, MA, USA) restriction sites (all primers used in this study are listed in Supporting information **Table S1**) and then transformed into the yeast strain YTK12 (Coolaber, Beijing, China). Transformants were grown on yeast minimal medium with sucrose in place of glucose at 30°C. To assay for invertase secretion, colonies were replica-plated on YPRAA plates containing raffinose and lacking glucose. The activity of invertase was further determined by monitoring the reduction of 2,3,5-triphenyltetrazolium chloride (TTC) (Sigma-Aldrich, MO, USA) dye to the insoluble red colored triphenylformazan (Oh et al., 2009). The yeast cell pellet was collected, washed, and resuspended in distilled sterile water, and an aliquot was incubated at 35°C for 35 min with 0.1% of the colorless dye TTC. Colorimetric changes were recorded after 5 min of incubation at room temperature. Three independent biological replicates were performed.

### 2.5 Bacterial T3SS-mediated overexpression in wheat plants

Candidate effector gene sequences were amplified using the Phusion high fidelity DNA polymerase (Thermo Scientific, MA, USA) according to the manufacturer’s instructions. All primers used in this study were designed using Primer Premier 5.0 (PREMIER Biosoft, Canada) and are listed in Supporting Information **Table S1**. The pEDV6: candidate effector recombinant plasmids were constructed using Gateway technology (Invitrogen, CA, USA) as described previously (Cheng et al., 2017). A stable T3SS delivery system of *P. fluorescens* has been developed and applied to deliver pathogen effectors into plant cells (Sohn et al., 2007; Thomas et al., 2009; Yin and Hulbert, 2011). The pEDV6: Sm10 and pEDV6: SmC002 vectors were constructed, and T3SS of *P. fluorescens* was then used to deliver candidate effectors in wheat leaves. The pEDV6: Sm10, pEDV6: SmC002 and pEDV6: AvrRpt2 (positive control) (Cheng et al., 2017) constructs and pEDV6 empty vector (negative control) were transformed into the *P. fluorescens* EtHAn strain by electroporation. The pEDV6 empty vector, pEDV6: AvrRpt2 and EtHAn strain were provided by Academician Dr. Zhensheng Kang from Northwest A & F University. Recombinant strains of EtHAn were grown in KB liquid medium for 48 h and resuspended in infiltration medium (10 mM MgCl_2_) after being washed two times using 10 mM MgCl_2_ (Sigma-Aldrich, MO, USA). EtHAn suspensions were infiltrated at an OD_600_ of 1.5 into the second leaves of wheat seedlings at the two-leaf stage (12-day-old) using a syringe without a needle. The infiltrated plants were grown and maintained in a cultivation room at 25°C for 2 days with a 16h: 8h light: dark cycle.

### 2.6 Detection of hydrogen peroxide in wheat leaves

Wheat leaves infiltrated with recombinant EtHAn carrying the empty vector or the bacterial avirulence protein AvrRpt2 were used as negative or positive controls, respectively. After 2 days of infiltration, the accumulation of hydrogen peroxide in wheat leaves was examined by staining with 1 mg/ml diaminobezidin (DAB) (Sigma-Aldrich, MO, USA) solution (10mmol/L Na_2_HPO_4_, pH3.8, Sigma-Aldrich, MO, USA) as described previously (Wang et al., 2007). The infiltration area of the wheat leaves was cut off, immediately immersed in DAB solution, and dyed for 24 hours in the dark. To visualize necrotic cell death, after washing with distilled water, leaf segments were fixed and destained in ethanol/acetic acid (1:1 v/v). The stained leaves were imaged using an Olympus SZX-16 (Olympus Corporation, Japan). Three independent biological replicates were performed.

### 2.7 Callose deposition in wheat leaves

For the visualization of callose, the leaves infiltrated with *P. fluorescens* EtHAn at 2 days were fixated and destained in 1: 3 acetic acid/ethanol (v/v) solution overnight. Callose deposition in the infiltrated leaves was detected using aniline blue diammonium salt solution (75 mmol/L K_2_HPO_4_, pH9.5, Sigma-Aldrich, MO, USA) according to the histochemical methods described by Hood and Shew (1996). Callose deposits were observed and photographed with an Echo Revolve Hybrid Microscope (Echo, USA) using a DAPI filter. Thirty sites (1.0 mm^2^/site) were selected randomly from infiltrated areas of each treated leaf, and the number of callose deposits was counted from each site using Echo Revolve Hybrid Microscope. Three independent biological replicates were conducted.

### 2.8 Total RNA extraction and RT-qPCR analysis

Total RNA from aphid *S. miscanthi* and wheat leaves was extracted with the TRIzol reagent (Invitrogen) according to the manufacturer’s protocols. After 6 hours starvation, five adults *S. miscanthi* were fed on healthy wheat for 0h, 6h, 12h, 24h, 48h, and then collected to characterize the transcript levels of *Sm10* and *SmC002* in *S. miscanthi* at different feeding stages. To detect the expression of glucan synthase genes and defense genes in wheat, leaf tissues from the infiltration sites of each plant were collected using sterilized scissors at 2 d and 4 d post inoculation. All of the samples were transferred to liquid nitrogen immediately and stored at −80°C until use. First-strand cDNA was synthesized from 1 μg RNA using TransScript One-Step gDNA Removal and cDNA Synthesis SuperMix (TransGen Biotech, Beijing, China). The *Sm10* and *SmC002* specific primers for qPCR were quoted from Zhang et al. (2017b). *β*-actin of aphid was used as internal reference gene (Yang et al., 2014). The marker gene of the phenylpropanoid pathway *phenylalanine ammonia-lyase* (*PAL*) and the induced salicylic acid (SA) marker protein *pathogenesis-related protein 1* (*PR-1*), and the genes tested for the jasmonic acid (JA)-responsive pathway included *lipoxygenase* (*LOX*) and *Ω-3 fatty acid desaturase* (*FAD*) were selected as target genes for the detection of wheat defense responses (Zhang et al., 2017a). Gene-specific primers for callose synthase genes were synthesized according to a previous study (Fu et al., 2014). The housekeeping gene *β-actin* of wheat was used as a reference (Liu et al., 2011; Zhang et al., 2017a). All primers are listed in **Supplementary Table S1**. RT-qPCR was performed using an ABI 7500 Real-Time PCR System (Applied Biosystems, CA, USA). cDNA was diluted 10-fold and used as the templates to examine the relative expression of genes in a 20 μL reaction system containing 2 μL of cDNA, 0.5 μL each of 10 μmol/L forward primer and reverse primer, 10 μL of 2× SYBR premix Ex Taq (Tli RNaseH Plus, Takara), and 0.4 μL of 50× ROX Reference Dye II (Tli RNaseH Plus, Takara) under the following conditions: 30 s at 95°C, followed by 40 cycles of 30 s at 95°C and 30 s at 60°C. The relative expression level of each gene was determined by using the 2^−ΔΔCT^ method (Livak and Schmittgen, 2001). There were three biological replicates for each treatment and time point.

### 2.9 Phytohormone analysis using LC-MS/MS

Quantifications of phytohormone levels were conducted according to a method described previously (Wu et al., 2007). In brief, at 2 d and 4 d post infiltration, approximately 500 mg infiltrated leaves from 10 individual plants with the same treatment were harvested and pooled to generate one biological replicate. Samples were homogenized in 1 mL acetonitrile (1% famic acid). Four nanogram of D4-SA, D6-JA, 1 ng JA-Ile-D6 to were added to the aqueous extraction solvent as internal standards. All samples were then vortexed for 2 min and centrifuged at 14,000 rpm for 10 min at 4°C. Eight hundred microlitres of supernatants was collected and evaporated to dryness using a vacuum concentrator to dry. Residues were resuspended in 200 µL of acetonitrile: H_2_O (1:1, v/v) and centrifuged at 14,000 g for 10 min. The supernatants were then collected and analyzed with a high-performance liquid chromatography-tandem mass spectrometry system (Agilent 1290 Infinity LC, QTRAP 5500, AB SCIES). Wheat leaves incubated with the pEDV6 empty vector were used as control groups. There were five biological replicates for each treatment and time point.

### 2.10 Aphid bioassay

After 2 days of infiltration, five alate adult *S. miscanthi* were transferred to infiltrated leaves within transparent plastic clip cages (2.7cm×2.7cm×2.7cm) to produce a population of age-synchronized nymphs. On the following day, all adults and other nymphs were removed, in order to avoid overcrowding and effects on plant defense responses, leaving only eight new-born nymphs on each treated leaf. The number of newly produced nymphs was recorded daily for a period of 14 days, and the nymphs were removed after each count to avoid overcrowding again. The number of surviving aphids was also recorded on the end day. After each 4-day period, the wheat plants were replaced by freshly infiltrated plants to guarantee continuous expression of effectors in the leaves. The pEDV6 empty vector was used as a control. A total of fifteen independent biological replicates were performed for each treatment.

### 2.11 Statistical analysis

All the data were analyzed using SPSS Statistics 20.0 software (RRID : SCR_002865) (http://www-01.ibm.com/software/uk/analytics/spss/), and the differences between or among groups were examined through independent sample t-test or one-way analysis of variance (ANOVA) LSD test. *P* values less than 0.05 were considered statistically significant.

## 3 Results

### 3.1 Sequence analysis of Sm10 and SmC002

**Figure 1:**
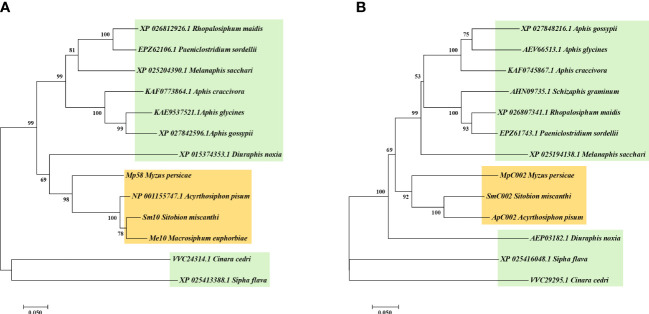
Phylogenetic analysis of Sm10 **(A)** and SmC002 **(B)** with the orthologs from other aphid species.

The two putative effector orthologs obtained from the salivary gland transcriptome of *S. miscanthi* (Zhang et al., 2017b) were designated Sm10 and SmC002. The open reading frames of these two candidate effectors were cloned and sequenced. We identified the homologous proteins of *S. miscanthi* using BLAST searches against the GenBank nonredundant (nr) protein database and performed phylogenetic analyses (**Figure 1**). Sm10 was orthologous to Me10 (*M. euphorbiae*), NP_001155747.1 (*A. pisum*), and Mp58 (*M. persicae*), sharing 76.3%–92.4% sequence identity. The similarity of SmC002 with ApC002 was 83.13% (which was the highest), followed by that with MpC002 (72.32%).

**Figure 2:**
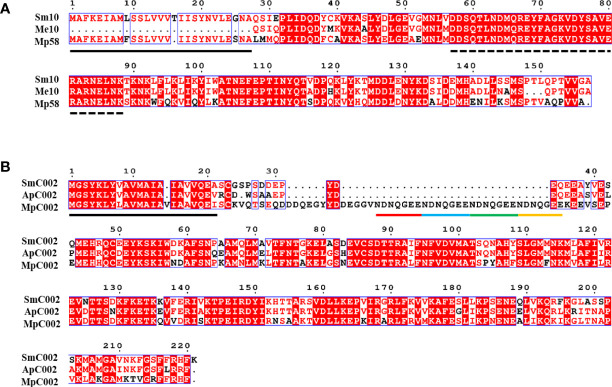
Multiple amino acid sequence alignments of two selected effectors of *Sitobion miscanthi* (Sm), and orthologs from *Acyrthosiphon pisum* (Ap), *Macrosiphum euphorbiae* (Me), and *Myzus persicae* (Mp). Conserved amino acids are highlighted with a red background colour. Predicted signal peptide sequences are underlined in black. **(A)** Sm10/Me10/Mp58 alignment. The dotted lines indicate the conserved domain between 57 and 87 amino acids. **(B)** SmC002/ApC002/MpC002 alignment. The four repeat motifs (NDNQGEE) in MpC002 are underlined with coloured lines.

All candidate effectors and homologous proteins had putative signal peptides at the N-terminal region (Me10 was a partial sequence), which is an important feature of the secretion of effectors (Kale, 2012). In the homologous proteins, the pfam domain, transmembrane helices, and nuclear localization signal were not found in the amino acid sequence, as assessed using bioinformatics analysis (**Table 1**). Amino acid sequence alignments revealed varying degrees of sequence divergence across the selected aphid effectors and proteins (**Figure 2**). Sm10 and its orthologs had a highly conserved domain within the 57–87 amino acid sequence region (**Figure 2A**). Compared with the amino acid sequence of MpC002, those of SmC002 and ApC002 lacked the NDNQGEE repeat in the N-terminal region (**Figure 2B**). Motifs with a high degree of conservation were detected and their positions in sequences were identified; these sites had a considerable impact on protein function and structure (**Supplementary Figure 1**).

**Table 1:** Sequence analyses of salivary effectors Sm10, Me10, Mp58, SmC002, ApC002 and MpC002.

Protein name	Number of amino acids (aa)	Molecular weight (kDa)	Signal peptide (aa)	Number of cysteines	Pfam domains	Transmembrane helices	Nuclear localization signal
Sm10	157	14.97	1-27	1	N	N	N
Me10	155	14.68	N	0	N	N	N
Mp58	127	17.85	1-26	1	N	N	N
SmC002	221	22.69	1-23	2	N	N	N
ApC002	219	25.19	1-23	2	N	N	N
MpC002	258	29.33	1-24	2	N	N	N

The number of cysteines is not included signal peptide. “N” represented the Pfam, transmembrance helices and nuclear localization signal were not found in the amino acid sequences.

### 3.2 Subcellular localization of Sm10 and SmC002 in *N. benthamiana*

**Figure 3:**
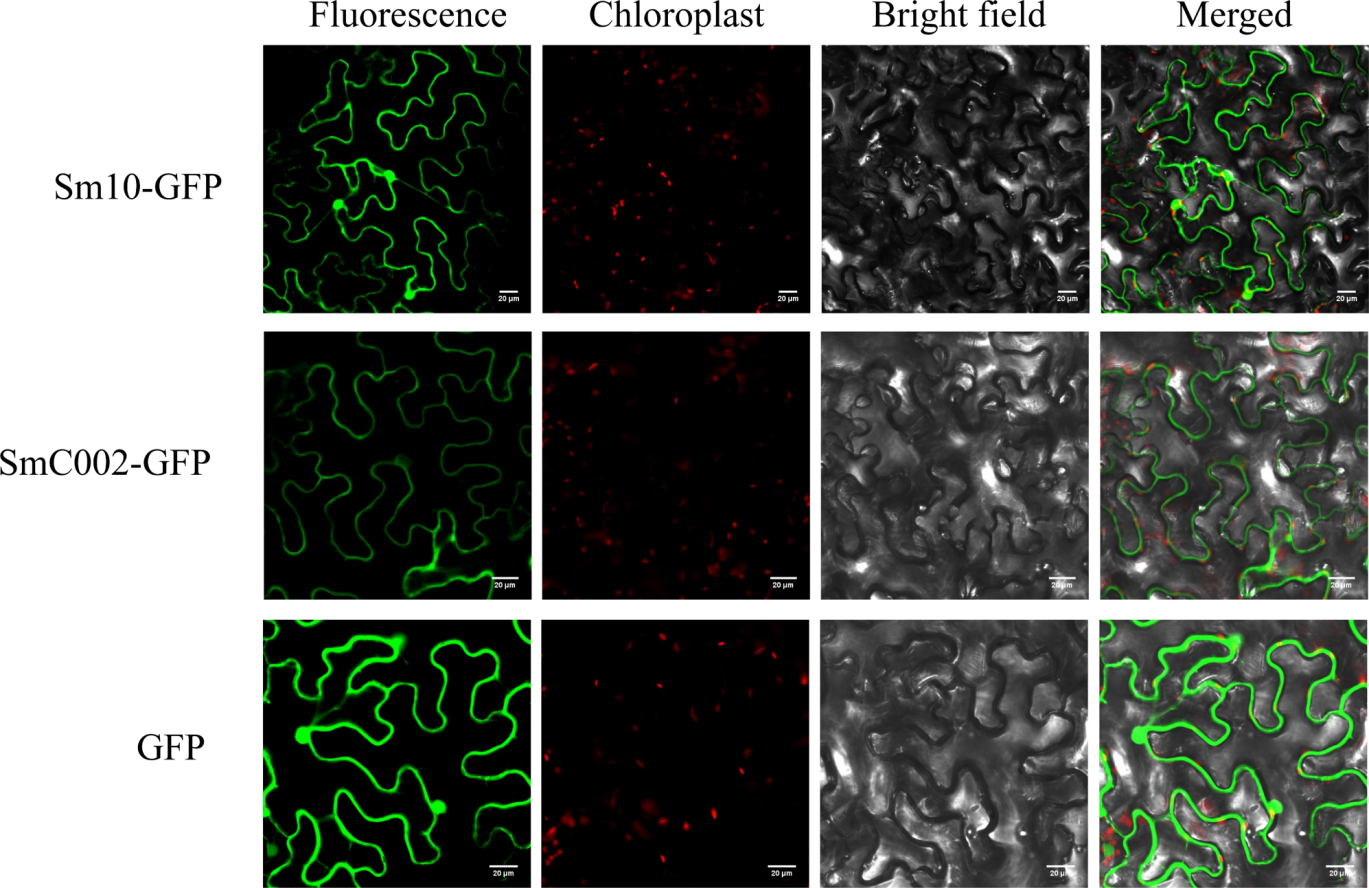
Subcellular localization of *Sm10 and SmC002* in *Nicotiana benthamiana* leaves. Candidate effector-GFP fusion protein and green fluorescent protein (GFP) (control) were expressed in *N. benthamiana* by transient agroinfiltration assays. The signals were observed and collected using a confocal microscope at 4 days after agroinfiltration. Scale bars= 20 μm.

To determine the subcellular localization of candidate effectors in the cellular compartment, effector-GFP fusion proteins (C-terminal GFP tag) were transiently expressed in the leaves of *N. benthamiana*. As shown in **Figure 3**, the control expressing GFP exhibited fluorescence throughout the whole cell (cytoplasm and nucleus), as revealed *via* confocal microscopy. Compared with the control, the fluorescence of SmC002-GFP fusion proteins was only observed in the plasma membranes of the epidermal cells of *N. benthamiana*. In contrast, the fluorescence of Sm10-GFP fusion proteins was detected in both the cytoplasm and nucleus in the leaves of *N. benthamiana*.

### 3.3 Transcript profiles of Sm10 and SmC002 in *S. miscanthi* during the aphid feeding stages

**Figure 4:**
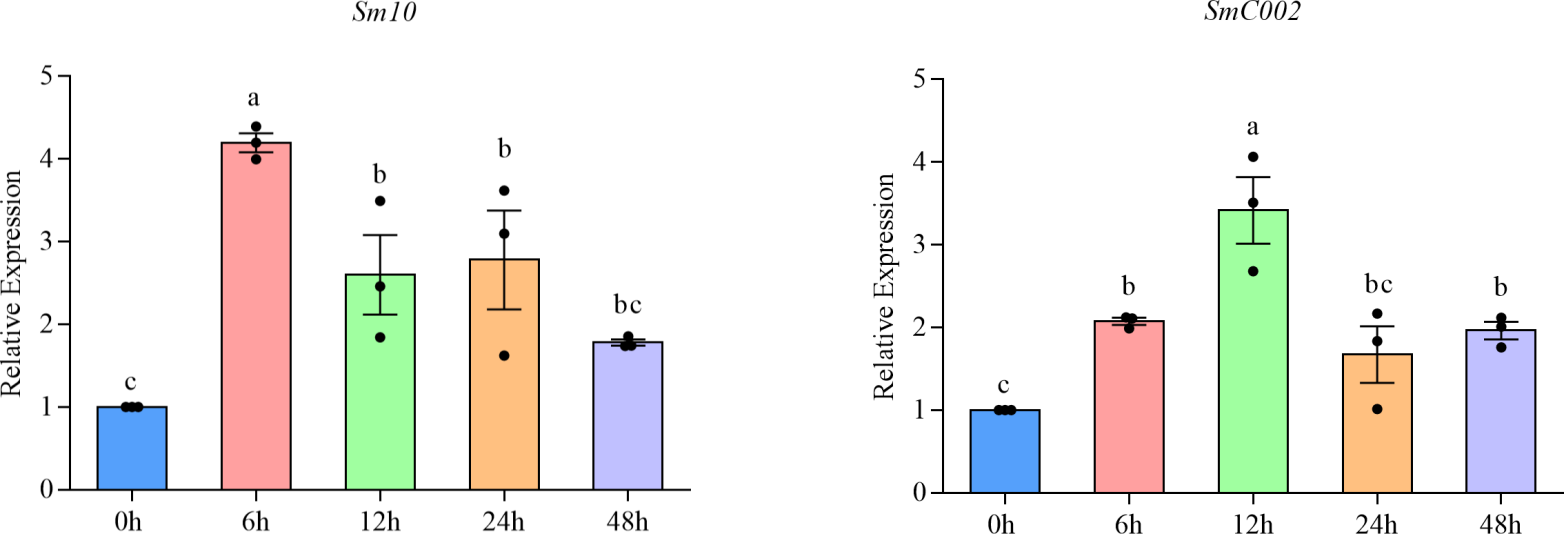
Relative expression levels of Sm10 and SmC002 of *S. miscanthi* after infestation on wheat plants at different time points. The values are presented as the means ± SE of three independent replicates. Different letters indicate significant differences according to one-way ANOVA LSD test (*P* < 0.05).

The transcript profiles of *Sm10* and *SmC002* in various feeding stages of *S. miscanthi* were characterized using qRT-PCR. The relative expression of *Sm10* and *SmC002* was differentially induced during the feeding of aphids on wheat and was found to peak at infection stages. The relative expression of *Sm10* peaked when aphids fed for 6 hours post-infection (hpi; 4.20-fold, *P* < 0.001), whereas that of *SmC002* peaked when aphids fed for 12 hpi (3.42-fold, *P* < 0.001). However, *Sm10* and *SmC002* exhibited no significant difference at 48 and 24 hpi, respectively (**Figure 4**). These results indicate that *Sm10* and *SmC002* are induced at the early feeding stage of aphids on wheat and may contribute to the interaction between aphids and plants.

### 3.4 Functional validation of the signal peptides of Sm10 and SmC002

The predicted signal peptide sequences of the Sm10 and SmC002 candidate effectors were cloned and fused into the vector pSUC2. As shown in **Supplementary Figure 2**, the negative control (Mg87) could not grow on YPRAA medium. In contrast, both Avr1b (a signal peptide of the Avr1b effector from *Phytophthora sojae*, which has been reported as a secretory leader and was used as the positive control) and two candidate effector-fused constructs enabled the invertase-mutated yeast strain to grow on CMD-W (YTK12 had the pSCU2 vector and showed growth without invertase secretion) and YPRAA (which allows growth only when invertase is secreted) media (**Supplementary Figure 2A**). Invertase secretion was further confirmed using an enzymatic activity test based on the reduction of TTC to the insoluble red-colored triphenylformazan. Both the candidate effector and Avr1b constructs enabled the conversion of TTC to the insoluble dark-red-colored triphenylformazan. However, the TTC-treated cultures remained almost colorless in the negative controls (the YTK12 and YTK12 yeast strains carrying Mg87; **Supplementary Figure 2B**).

These results confirmed the secretory functions of the putative N-terminal signal peptides of the candidate effectors Sm10 and SmC002, which were secreted from *S. miscanthi*. Moreover, Sm10 (LOC100167427) and SmC002 (LOC100167863) were previously detected in the saliva proteomes of *S. miscanthi* (Zhang et al., 2021), which further suggests that Sm10 and SmC002 can be secreted into plant cells by aphids.

### 3.5 Delivery of Sm10 and SmC002 into wheat had no effect on plant phenotype and hydrogen peroxide accumulation

**Figure 5:**
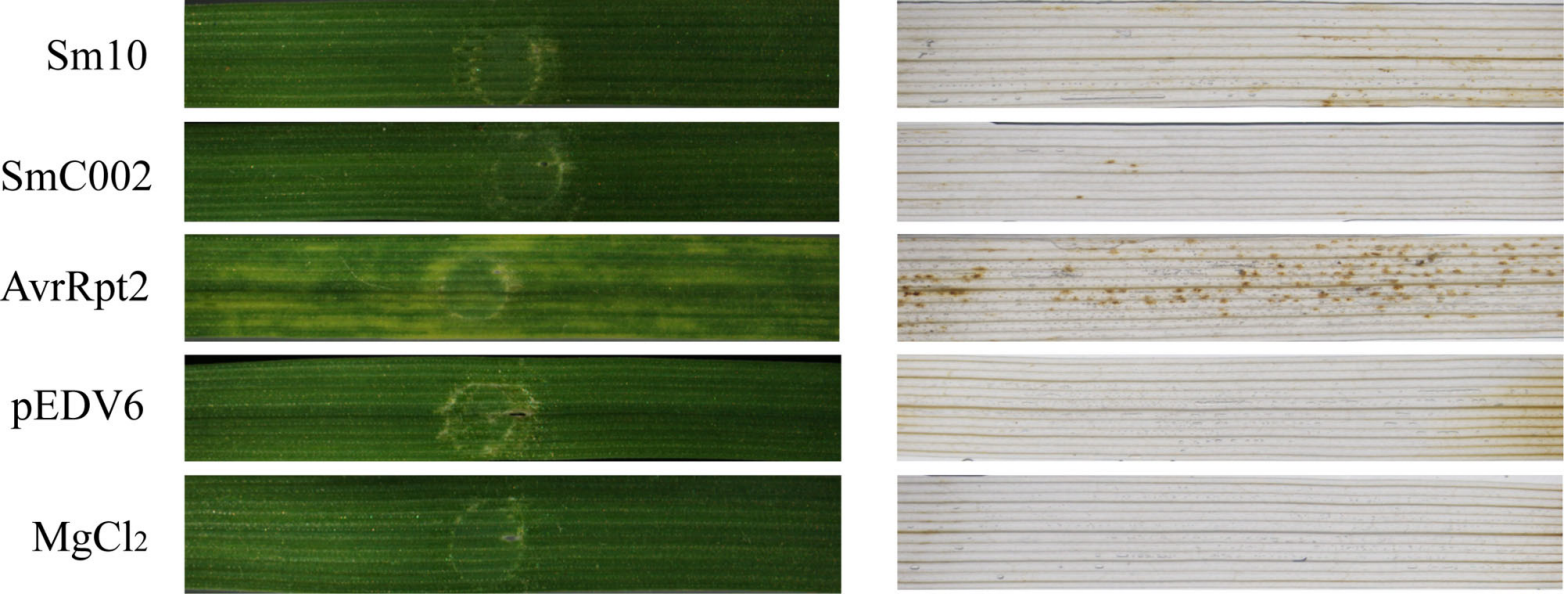
Effects of delivering Sm10 and SmC002 into wheat on leaf phenotypes and hydrogen peroxide (H_2_O_2_) accumulation. The left is phenotypes of wheat leaves inoculated with pEDV6: Sm10 and pEDV6: SmC002 at 4 days. The right is detection of H_2_O_2_ accumulation in wheat leaves inoculated with pEDV6: Sm10 and pEDV6: SmC002 at 4 days using DAB staining. Leaves infiltrated with MgCl_2_ buffer and EtAnH carrying the pEDV6 empty vector were used as a negative control. Leaves infiltrated with EtAnH carrying the effector AvrRpt2 from *Pseudomonas syringae* were used as a positive control. Three biological replicates were performed. Similar results were observed when these experiments were performed in duplicate.

A bacterial type III secretion system (T3SS) including the expression vector pEDV6 and the *P. fluorescens* EtHAn strain was used to deliver the effectors of *S. miscanthi* into wheat and investigate their functions. The AvrRpt2-inoculated wheat plants (positive control) displayed obvious chlorotic reaction phenotypes at 4 days, and DAB staining results revealed high levels of H_2_O_2_ accumulation in the infiltration areas, indicating that the T3SS expression/delivery system was effective on wheat. In contrast, inoculation of the EtHAn strain carrying the empty vector pEDV6 into wheat did not result in any phenotypic changes or H_2_O_2_ accumulation. Moreover, infiltration of the EtHAn strain with the pEDV6:Sm10 and pEDV6:SmC002 constructs also did not induce any obvious chlorosis symptoms or H_2_O_2_ accumulation in the infiltrated regions (**Figure 5**).

### 3.6 Delivery of Sm10 and SmC002 suppressed PTI-associated callose deposition in wheat

**Figure 6:**
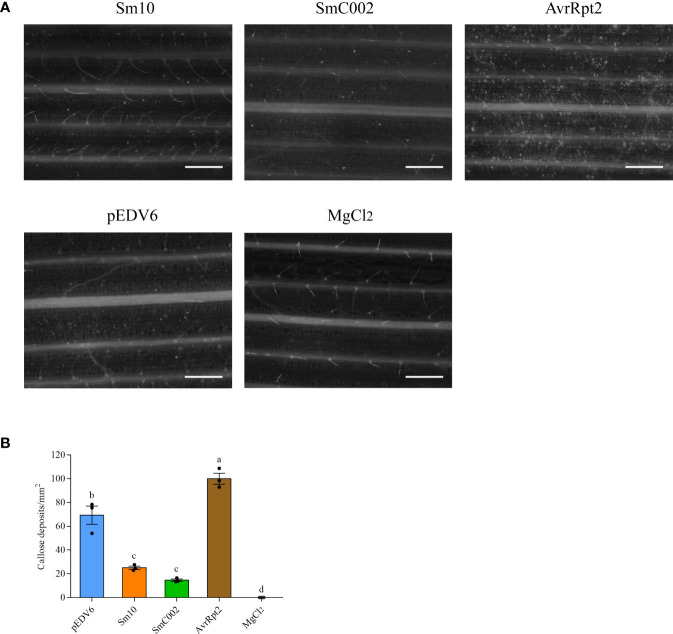
Callose deposition in wheat leaves expressing Sm10 and SmC002. **(A)** Aniline blue staining was performed to examine callose deposition in wheat leaves infiltrated with EtAnH carrying pEDV6: Sm10 and pEDV6: SmC002 at 4 days using epifluorescence microscopy. **(B)** Average number of callose deposits per mm^2^ in wheat leaves inoculated with pEDV6: Sm10, and pEDV6: SmC002 at 4 days. The values are presented as the means ± SE of three independent replicates. Different letters indicate significant differences according to analysis of variance (ANOVA, *P* < 0.05). Leaves infiltrated with MgCl_2_ buffer and EtAnH carrying the pEDV6 empty vector were used as negative controls. Leaves infiltrated with EtAnH carrying the effector pEDV6: AvrRpt2 was used as a positive control. Bar=0.33mm.

Callose deposition is regarded as a response associated with the later stages of PTI. Aniline blue staining showed that callose deposition was present on wheat plants inoculated with EtHAn and carrying the empty pEDV6 vector (control group), indicating that infection with nonpathogenic EtHAn triggered PTI in wheat (**Figure 6A**). Compared with the control group, a lower amount of callose deposition was observed in wheat leaves inoculated with Sm10 and SmC002 (**Figures 6A, B**), and the number of callose deposits in wheat leaves significantly reduced after the delivery of Sm10 and SmC002 (*P* < 0.001) compared with AvrRpt2 (positive control) and pEDV6. These results indicated that Sm10 and SmC002 inhibited callose deposition and PTI.

### 3.7 Sm10 and SmC002 affected the expression levels of glucan synthase genes

**Figure 7:**
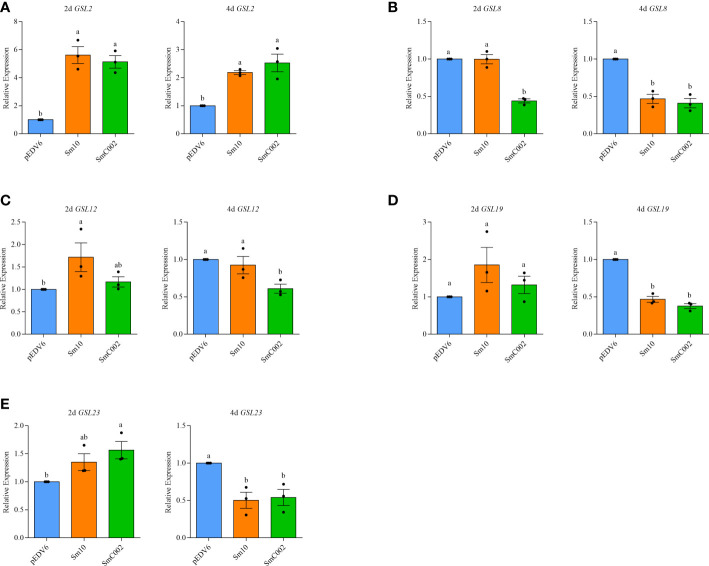
Relative expression levels of callose synthase genes in wheat leaves infiltrated with EtAnH carrying pEDV6: Sm10 and pEDV6: SmC002 at 2 and 4 days post infiltration. **(A)** Relative expression levels of *GLS2*. **(B)** Relative expression levels of *GLS8*. **(C)** Relative expression levels of *GLS12*. **(D)** Relative expression levels of *GLS19*. **(E)** Relative expression levels of *GLS23*. *β-actin* of wheat was used as a reference gene. The values are presented as the means ± SE of three independent replicates. Different letters indicate significant differences among treatments (ANOVA, *P* < 0.05).

Glucan synthases are key enzymes involved in the biosynthesis of callose. To verify the effects of the effectors on wheat callose deposition, the expression levels of five glucan synthase genes were examined using RT-qPCR (Fu et al., 2014). Our results showed that the expression levels of *GSL2* (5.13- to 5.61-fold, *P* = 0.001) and *GSL12* (1.72-fold; *P* < 0.05) in Sm10-delivered wheat leaves and of *GSL23* (1.56-fold; *P* < 0.05) in SmC002-delivered wheat leaves were significantly upregulated compared with those detected in the controls. However, the transcript levels of *GSL8* (0.41- and 0.47-fold, *P* < 0.001), *GSL19* (0.38- and 0.47-fold, *P* < 0.001), and *GSL23* (0.50- and 0.54-fold, *P* < 0.05) in wheat leaves were significantly decreased at 4 days post-infiltration of EtAnH carrying Sm10 and SmC002 (**Figures 7A–E**). These results further suggested that Sm10 and SmC002 inhibited leaf callose accumulation by affecting the expression of glucan synthase genes.

### 3.8 Sm10 and SmC002 induced the SA -dependent defense signaling pathway

**Figure 8:**
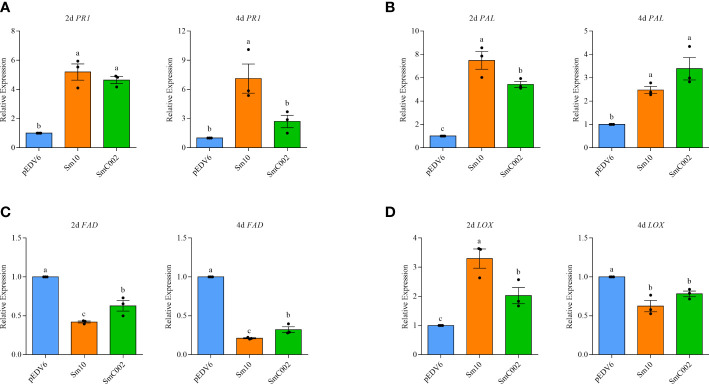
Relative expression of salicylic acid (SA) and jasmonic acid (JA) signalling-related genes in wheat leaves expressing Sm10 and SmC002 at 2 and 4 days. **(A, B)** Relative expression of *PR1* and *PAL* genes which involved in the SA defense pathway. **(C, D)** Relative expression of *FAD* and *LOX* genes associated with the JA defense pathway. *β*-*actin* of wheat was used as a reference gene. The values are presented as the means ± SE of three independent replicates. Different letters indicate significant differences according to one-way ANOVA LSD test (*P* < 0.05).

**Figure 9:**
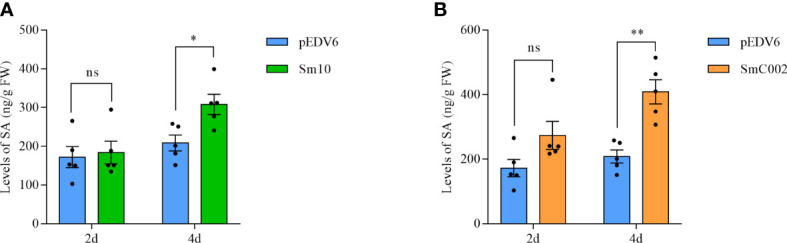
Levels of salicylic acid (SA) in wheat leaves inoculated with *S. miscanthi* candidate effectors at 2 and 4 days post infiltration of EtAnH. **(A)** Levels of SA in wheat leaves infiltrated with EtAnH carrying Sm10 at 2 and 4 days post infiltration. **(B)** Levels of SA in wheat leaves infiltrated with EtAnH carrying SmC002 at 2 and 4 days post infiltration The values are presented as the means ± SE of five independent replicates. *P < 0.05, **P < 0.05 based on the Student’s t-test. ns, no significant difference.

To further investigate the roles of Sm10 and SmC002 in the regulation of plant defenses, the relative expression of key genes associated with SA and JA and the levels of plant defense hormones in treated wheat leaves were examined. The results showed that the transcript levels of the SA biosynthesis enzyme *PAL* (2.5- to 7.5-fold) were significantly upregulated after treatment with Sm10 and SmC002 at 2 days (*P* < 0.001) and 4 days (*P* = 0.003) and that the expression levels of *PR1* (4.6- to 5.2-fold) were significantly increased at 2 days compared with the control (*P* < 0.001; **Figures 8A, B**). Furthermore, delivery of Sm10 and SmC002 into wheat significantly increased the levels of SA at 4 days compared with the control (**Figures 9A, B**), indicating that these two effectors can induce the SA signaling pathway in wheat.

The expression levels of *LOX* (2.02- to 3.30-fold, *P* = 0.002) were significantly upregulated at 2 days after SmC002 or Sm10 infiltration. However, the expression levels of *FAD* (0.21- to 0.32-fold, *P* < 0.001) and *LOX* (0.62- to 0.78-fold, *P* = 0.004), which are involved in the JA signaling pathway, were significantly reduced at 4 days post-infiltration of the two candidate effectors (**Figures 8C, D**). JA and JA-isoleucine were not detected in our study.

### 3.9 Sm10 and SmC002 promoted aphid performance on wheat

**Figure 10:**
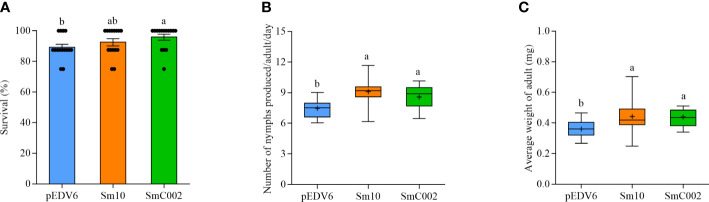
Survival and fecundity of *S. miscanthi* after feeding on wheat leaves delivering with Sm10 and SmC002. **(A)** Survival of *S. miscanthi* on wheat leaves expressing candidate effectors. The values are presented as the means ± SE. **(B)** Number of nymphs produced per *S. miscanthi* adult on wheat plants expressing candidate effectors. Box plots show the average number of nymphs per adult/day after feeding on infiltrated areas of wheat leaves incubated with candidate effectors for 14 days. **(C)** Average weight of *S. miscanthi* adult on wheat plants expressing candidate effectors. Wheat leaves incubated with the pEDV6 empty vector were used as a control. The bars show the maximum and minimum values, + present mean values. Fifteen biological replicates were performed for each treatment. Different letters indicate significant differences according to one-way ANOVA LSD test (*P* < 0.05).

To investigate the roles of these two putative effectors in the modulation of host susceptibility, the performance of aphids was determined when the aphids were allowed to feed on wheat leaves incubated with EtHAn carrying the effector. As shown in **Figure 10A**, the survival of *S. miscanthi* feeding on SmC002-expressing leaf areas (95.83% ± 4.45%) was significantly higher than that of *S. miscanthi* feeding on empty vector (control)-infiltrated areas (89.17% ± 4.62%). In addition, the number of nymphs produced by adults and the average weight of adults were both significantly higher in aphids feeding on Sm10- and SmC002-treated leaves (*P* = 0.001; *P* = 0.006; **Figures 10B, C**). These results suggested that Sm10 and SmC002 enhanced aphid performance and host susceptibility.

## 4 Discussion

The grain aphid *S. miscanthi* is an important and globally distributed cereal aphid, causing yield losses and transmitting barley yellow dwarf virus (Blackman and Eastop, 2000). C002, the first salivary protein identified, plays an essential role in feeding on a host plant (Mutti et al., 2008), and the overexpression of Me10 can modulate the susceptibility of host plants to aphids (Atamian et al., 2013), these two effectors have not yet been characterized in *S. miscanthi*. The amino acid sequence is conserved between effectors and homologous proteins, but effectors impact the outcome of plant-aphid interactions differently depending on different host species (Schulze-Lefert and Panstruga, 2011; Kettles and Kaloshian, 2016). We found that Sm10 and SmC002 were both highly induced during early feeding stages of aphids on wheat plants, it indicated that they may be involved in regulating the interaction between *S. miscanthi* and host plants.

Consistent with previous studies, MpC002 from *M. persicae* and RpC002 from *R. padi* have been demonstrated to promote plant susceptibility to aphids in a species-specific manner (Pitino and Hogenhout, 2013; Escudero-Martinez et al., 2020). SmC002 and MpC002 were both localized at the plasma membrane in *N. benthamiana* (Escudero-Martinez et al., 2020), indicating that this could be the site of activity for these effectors. These results pointed to a potentially conserved function of C002, but the underlying mechanism remains to be elucidated. We also found that the NDNQGEE repeat motif was absent in SmC002, and this repeat motif was previously demonstrated to be associated with the *M. persicae* virulence; overexpression of the MpC002 mutant without the NDNQGEE repeat region does not promote *M. persicae* colonization in *Arabidopsis* (Pitino and Hogenhout, 2013). The NDNQGEE repeat regions were also lacking in ApC002 of *A. pisum* and RpC002 of *R. padi* (Mutti et al., 2008; Escudero-Martinez et al., 2020). *M. persicae*, a generalist aphid species, can feed on a wide range of host plants (Weber, 1986; Blackman and Eastop, 2000), but *S. miscanthi*, *A. pisum* and *R. padi* are oligophagous (Goławska and Lukasik, 2012; Hu et al., 2015; Jiang et al., 2019), therefore, whether NDNQGEE repeat regions are related to the range of aphid host plants remains to be elucidated. It is also noteworthy that the numbers of the NDNQGEE repeat were highly divergent from those of the *M. persicae* genotypes (Thorpe et al., 2016). The biological significance of this repeat variation also needs to be further investigated.

The type III secretion system (T3SS) of *P. fluorescens* has been maturely applied to the study of the interaction model between pathogen or grain aphid effectors with wheat, and can successfully secrete candidate effectors stably into wheat leaf cells (Li et al., 2022; Liu et al., 2022; Zhang et al., 2022). In this study, we did not directly demonstrate that effectors are delivered in leaf cells, but wheat leaves infiltrated with EtHAn carrying AvrRpt2 exhibited a noticeable chlorotic phenotype and H_2_O_2_ production through the infiltrated region that was not observed in wheat seedlings infiltrated with EtHAn carrying an empty pEDV6 vector, the similar results with previous studies (Yin and Hulbert, 2011; Cheng et al., 2017; Zhang et al., 2022), indicating that effector proteins were delivered effectively into wheat by the *P. fluorescens* delivery system. Compared with the positive control, no obvious phenotype or H_2_O_2_ accumulation was detected after delivery of Sm10, SmC002 into wheat leaves, suggesting that no HR was induced by these two effectors. Infestation of the aphids, such as *S. avenae*, *M. persicae* and the greenbug *S. graminum*, results in callose deposition in host plants, which contributes to the aphid resistance (Li et al., 2018; Zhang et al., 2019; Yao et al., 2020). We found that overexpression of Sm10 and SmC002 in wheat leaves led to the inhibition of PTI-related callose deposition. The expression levels of callose synthase genes (*GSL8*, *GSL19* and *GSL23*) were also significantly reduced in wheat leaves inoculated with Sm10 and SmC002. These results suggested that Sm10 and SmC002 are involved in the suppression of wheat PTI by inhibiting callose synthesis. However, we found that delivering the Sm10 and SmC002 into wheat leaves increased the expression levels of SA defense genes and the levels of SA. Previous study demonstrated that callose deposition is dependent on SA accumulation (Donofrio and Delaney, 2001), and reduced callose formation is observed in *SA*-deficient NahG transgenic potato plants after infection with *Phytophthora infestans* (Halim et al., 2007). However, Tomczynska et al. (2020) found that the RxLR3 effector of the *Arabidopsis* pathogen *Phytophthora brassicae* can directly interacts with a subgroup of callose synthases and had a negative effect on callose deposition. But, the mechanisms of aphid salivary effectors on the suppression of callose production are still unknown.

Compared with SA-mediated defense responses, the JA-dependent defense pathway is proposed to be effective in plant resistance to aphids Züst and Agrawal, 2016. Previous studies showed that spraying SA on wheat seedlings had no significant effects on *S. avenae* performance, while exogenous application of JA on *Arabidopsis*, sorghum, legume, wheat and tomato plants had negative effects on aphid population growth (Strauss, 1991; Bruce et al., 2003; Gao et al., 2007; Zhang et al., 2017a). It has been hypothesized that phloem-feeding insects can manipulate the crosstalk between SA-JA signalling networks to manipulate plant defenses for their own benefit (Pieterse and Dicke, 2007; Walling, 2008). Xu et al. (2019) found that salivary effector Bt56 of whitefly *Bemisia tabaci* promotes susceptibility of tobacco to the whitefly by eliciting SA-mediated plant defenses. Consistently, we also found that the expression levels of JA-responsive genes were downregulated in wheat leaves infiltrated with EtHAn carrying these two effectors, and the levels of JA and JA-Ile were failed to be detected because of too low levels. It is speculated that Sm10 and SmC002 enhance host susceptibility by inducing the SA pathway to suppress more detrimental JA defenses. It is worthy to screen the target proteins of Sm10 and SmC002 in wheat leaves using yeast two-hybrid system in the future study to figure out the its mechanisms on the regulation of plant immunity. Also, aphids mainly feed from plant vascular tissue and secrete effectors into the vasculature. Previous study demonstrated that *M*. *persicae* effector Mp1 promotes virulence on *N*. *benthamiana* upon phloem-specific overexpression (Rodriguez et al., 2017). Therefore, it needs to achieve specific expression of aphid salivary effectors in plant phloem *via P. fluorescens* EtHAn-mediated delivery systems in the future study.

## 5 Conclusion

In this study, two putative salivary effectors, Sm10 and SmC002, were delivered into wheat cells *via* the type III secretion system (T3SS) of *P. fluorescens*. Our findings demonstrated that Sm10 and SmC002 induced the SA-dependent defense signaling pathway, whereas they inhibited PTI-associated callose deposition, resulting in the promotion of host susceptibility. These results provided further insights into the mechanisms underlying cereal aphid interactions with wheat. In addition, the *P. fluorescens* delivery system was shown to be a useful method for the large-scale screening of candidate effectors from cereal aphids.

## Data availability statement

The datasets presented in this study can be found in online repositories. The names of the repository/repositories and accession number(s) can be found in the article/**Supplementary Material**.

## Author contributions

YF, YZ and JC conceived and designed the experiments. YF, XL and QW performed the experiments, YF, HLiu and YC analyzed the data. YF and YZ write the manuscript. YZ, HLi and JC revised the manuscript. All authors contributed to the article and approved the submitted version.
