# Supplementary material for: Two salivary proteins Sm10 and SmC002 from grain aphid Sitobion miscanthi modulate wheat defense and enhance aphid performance
Source: Front Plant Sci. 2023 Mar 28;14:1104275. doi: 10.3389/fpls.2023.1104275 (PMC10086322; doi:10.3389/fpls.2023.1104275)
Supplement: Supplementary file 1 [file DataSheet_1.docx]

**Supplementary Figure Legends**

**Supplementary Figure 1** Motif prediction of Sm10 and SmC002 with the orthologs from *Acyrthosiphon pisum* (Ap), *Macrosiphum euphorbiae* (Me), and *Myzus persicae* (Mp).

**Supplementary Figure 2** Functional validation of the signal peptides of Sm10 and SmC002. **(A)** Functional validation of the signal peptides of Sm10 and SmC002 was performed using the yeast invertase secretion assay. Yeast YTK12 strains carrying the signal peptide fragments fused in frame to the invertase gene in the pSUC2 vector were able to grow in both CMD-W and YPRAA media. **(B)** The 2,3,5-triphenyltetrazolium chloride (TTC) colour reaction was used to verify the functional validation of the signal peptides. Yeast YTK12 strains carrying the signal peptide fragments fused in frame to the invertase gene in the pSUC2 vector reduced TTC to the red coloured 1,3,5-triphenylformazan (TPF). The untransformed YTK12 strain and the first 25 amino acids of the non-secreted Mg87 protein from *Magnaporthe oryzae* were used as negative controls, and the signal peptide of the effector Avr1b from *Phytophthora sojae* was used as a positive control.
