# Supplementary material for: Two salivary proteins Sm10 and SmC002 from grain aphid Sitobion miscanthi modulate wheat defense and enhance aphid performance
Source: Front Plant Sci. 2023 Mar 28;14:1104275. doi: 10.3389/fpls.2023.1104275 (PMC10086322; doi:10.3389/fpls.2023.1104275)
Supplement: Supplementary file 2 [file Table_1.docx]

**Table S1.** Oligonucleotides used in the study.

| **No** | **Name** | **Sequences (5' to 3’)** | **Purpose** |
| --- | --- | --- | --- |
| 1 | pSUC2-*Sm10*-F | ccggaattcATGGCTTTCAAGGAAATTG | Insertion of Sm10 signal peptide into pSUC2 vector |
| 2 | pSUC2-*Sm10*-R | ccgctcgagTGCATTGCCTTCTAGTACATT |  |
| 3 | pSUC2-*SmC002*-F | ccggaattcATGGGAAGTTACAAATTATACG | Insertion of SmC002 signal peptide into pSUC2 vector |
| 4 | pSUC2-*SmC002*-R | ccgctcgagGCAACTAGCTTCCTGTACTACA |  |
| 9 | pSUC2-F | TCCTCGTCATTGTTCTCGTTCC | Primer of pSUC2 vector |
| 10 | pSUC2-R | TAATACGACTCACTATAGGG |  |
| 11 | *β-Actin*-F | CGTTACCAACTGGGACGATATG | qRT-qPCR analysis of reference *Actin* in *Sitobion miscanthi* |
| 12 | *β-Actin*-R | GGGTTCAATGGAGCTTCTGTTA |  |
| 13 | *Sm10*-F | GGTGGTGGTCACTATCAT | qRT-PCR analysis of *Sm10* in *Sitobion miscanthi* |
| 14 | *Sm10*-R | TTCATTCCAACTTCTCCC |  |
| 15 | *SmC002*-F | GGTAAAGAATTAGCCTCCG | qRT-PCR analysis of *SmC002* in *Sitobion miscanthi* |
| 16 | *SmC002*-R | GTCGTGTTCACCTCCCTC |  |
| 17 | attB-*Sm10*-F | ggggacaagtttgtacaaaaaagcaggcttcCAATCAATAGAACCATTAATAG | Insertion of *Sm10* sequence into pEDV6 vector |
| 18 | attB-*Sm10*-R | ggggaccactttgtacaagaaagctgggtcTTAAGCTCCAACGACTGT |  |
| 19 | attB-*SmC002*-F | ggggacaagtttgtacaaaaaagcaggcttcGGTAGTCCGTCTGACGAT | Insertion of *SmC002* sequence into pEDV6 vector |
| 20 | attB-*SmC002*-R | ggggaccactttgtacaagaaagctgggtcTTATTTAAAATGTCGAAAGAA |  |
| 21 | *M13*-F | GTAAAACGACGGCCAGT | Primer of pEDV6 vector |
| 22 | *M13*-R | CAGGAAACAGCTATGAC |  |
| 23 | *GSL2*-F | CTGGGAGTGCTGGTGCTGAT | qRT-PCR analysis of *GSL2* in wheat |
| 24 | *GSL2*-R | TAACAATCACTCCAAGCAGTATCTC |  |
| 25 | *GSL8*-F | TCTATGTGACTACTGTTGGGTT | qRT-PCR analysis of *GSL8* in wheat |
| 26 | *GSL8*-R | CACGCTCAATGCTTTATTT |  |
| 27 | *GSL12*-F | GTTCTTCTCGTGGTTCCCCTTT | qRT-PCR analysis of *GSL12* in wheat |
| 28 | *GSL12-*R | GTCCCTAATCAAGTCCAGAAATGTA |  |
| 29 | *GSL19*-F | TCATCCCAACTGTCTGGTTTATT | qRT-PCR analysis of *GSL19* in wheat |
| 30 | *GSL19*-R | TATCTACGGAGCACAGCCCCACT |  |
| 31 | *GSL23*-F | AACCTCCGCCTTCCGACAGA | qRT-PCR analysis of *GSL23* in wheat |
| 32 | *GSL23*-R | GCATACAGGAACACTCGGAATC |  |
| 33 | *PR1*-F | AGTGCAAGTCCACCCTCATC | qRT-PCR analysis of *PR1* in wheat |
| 34 | *PR1*-R | CTTGGCCTTGGTGATCTCAT |  |
| 35 | *PAL-*F | CCACCCTGGACAGATTGAA | qRT-PCR analysis of *PAL* in wheat |
| 36 | *PAL*-R | ATGAGCGGGTTGTCGTTG |  |
| 37 | *LOX*-F | GACCAGCGAAACAACAACC | qRT-PCR analysis of *LOX* in wheat |
| 38 | *LOX*-R | GCATACAATAGCGGGAACAC |  |
| 39 | *FAD*-F | TCCCATTCCACCTACTGC | qRT-PCR analysis of *FAD* in wheat |
| 40 | *FAD*-R | GGACTCACCAATCCGAGA |  |
| 41 | *β-Actin*-F | GGAAAATCAGTCTCGGTTCAG | qRT-PCR analysis of reference *Actin* in wheat |
| 42 | *β-Actin*-R | TCATACAGCAGGCAAGCAC |  |
| 43 | pBWA(V)HS-*Sm10* GLosgfp-F | gcaggctcaggggatatcCAATCAATAGAACCATTAATAG | Insertion of *Sm10* sequence into pBWA(V)HS vector |
| 44 | pBWA(V)HS-*Sm10* GLosgfp-R | cagggcgatatcgatatcAGCTCCAACGACTGTT |  |
| 45 | pBWA(V)HS-*SmC002*-GLosgfp-F | gcaggctcaggggatatcGGTAGTCCGTCTGACGAT | Insertion of *SmC002* sequence into pBWA(V)HS vector |
| 46 | pBWA(V)HS-*SmC002*-GLosgfp-R | cagggcgatatcgatatcTTTAAAATGTCGAAAGAA |  |

Notes: The capital letters indicate bases that match the initial template. The lowercase letters indicate the added sequences including restriction enzyme sites and *attB* sites into the PCR products.
