# Supplementary figures and images for: Two salivary proteins Sm10 and SmC002 from grain aphid Sitobion miscanthi modulate wheat defense and enhance aphid performance
Source: Front Plant Sci. 2023 Mar 28;14:1104275. doi: 10.3389/fpls.2023.1104275 (PMC10086322; doi:10.3389/fpls.2023.1104275)

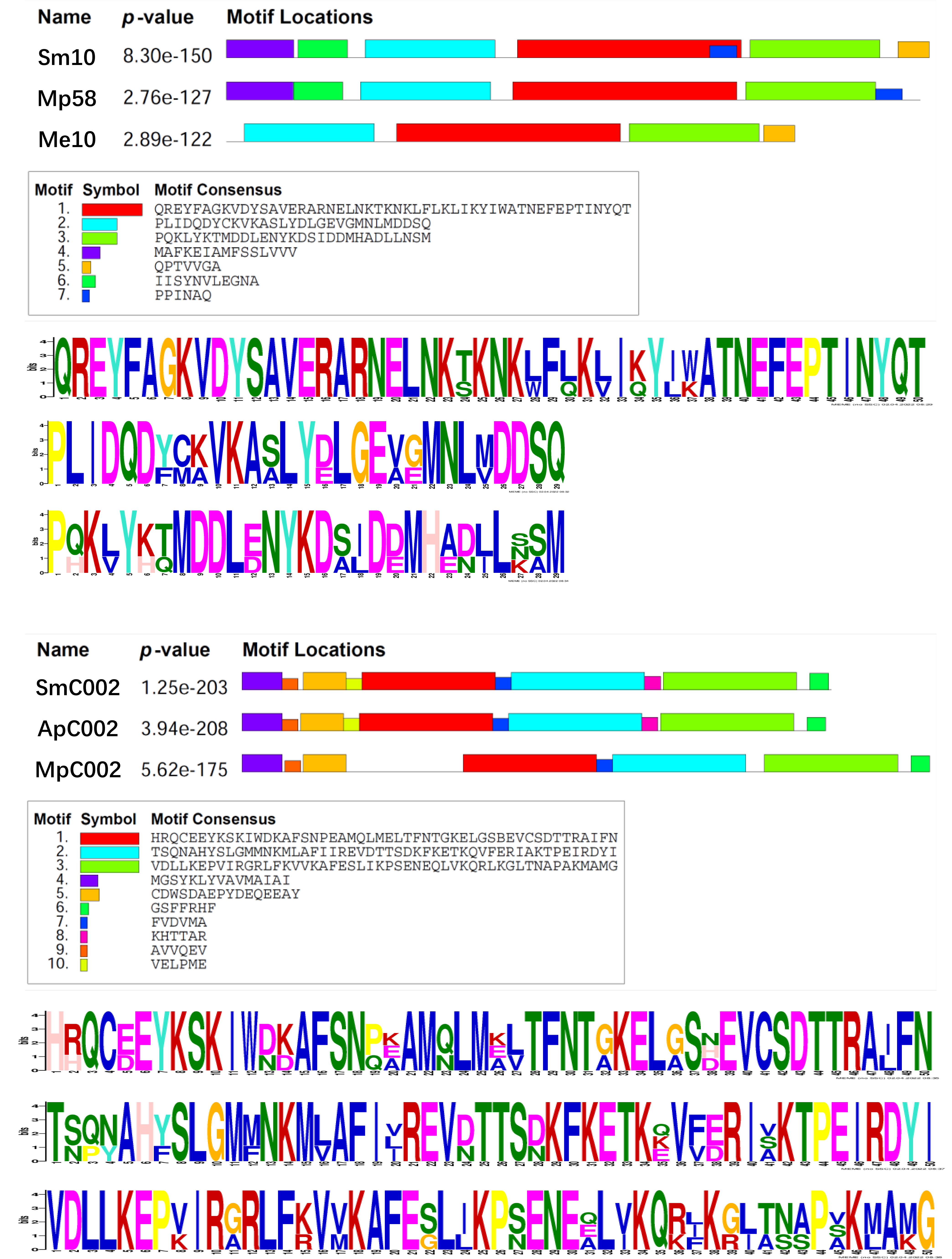

Supplement: Supplementary file 3 [file Image_1.tif]

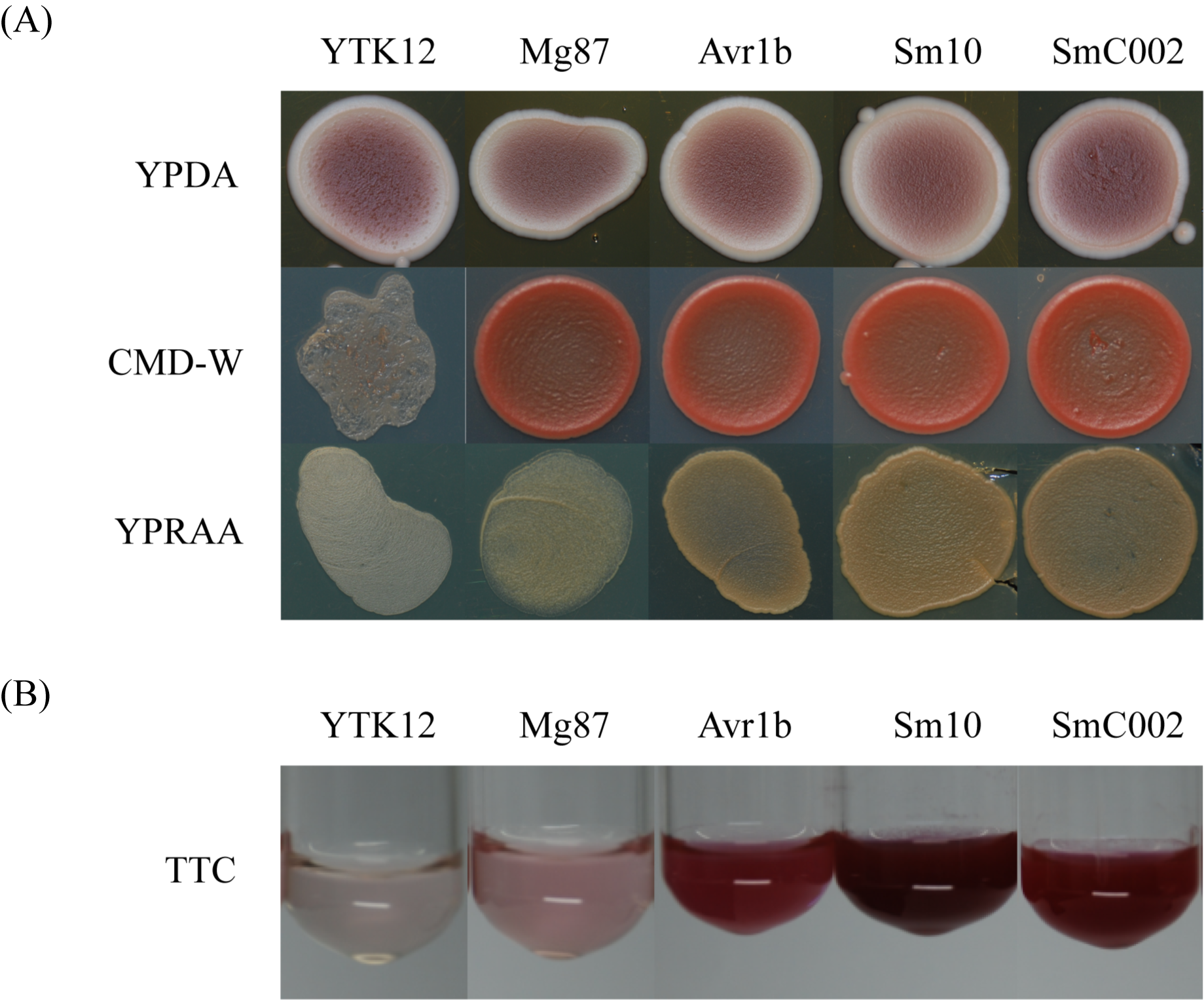

Supplement: Supplementary file 4 [file Image_2.tif]
